# O-GlcNAcylation in novel regulated cell death: ferroptosis, pyroptosis, and necroptosis

**DOI:** 10.1038/s41420-025-02895-x

**Published:** 2025-12-05

**Authors:** Ying-Zi Wang, Hao-Yu Zhao, Tashi Nyima, Zhaowu Ma

**Affiliations:** 1 The First Affiliated Hospital of Yangtze University, Yangtze University, Jingzhou, Hubei China; 2 School of Basic Medicine, Health Science Center, Yangtze University, Jingzhou, Hubei China; 3 Naidong District People’s Hospital, Shannan, Tibet Autonomous Region, Shannan, China

**Keywords:** Glycobiology, Glycomics, Glycosylation, Glycosylation

## Abstract

GlcNAcylation, a dynamic post-translational modification involving the addition of N-acetylglucosamine to serine and threonine residues, has emerged as a key regulatory factor in cellular metabolism and signaling. Ferroptosis, pyroptosis, and necroptosis are newly discovered forms of regulated cell death that play crucial roles in various physiological and pathological processes, including cancer development, neurodegeneration, and inflammation. This review aims to summarize the functions of O-GlcNAcylation in modulating these distinct cell death pathways, with a focus on their implications in disease mechanisms and potential therapeutic applications. We summarize the mechanisms by which O-GlcNAcylation modulates ferroptosis, pyroptosis, and necroptosis, and explore the potential of targeting O-GlcNAcylation as a promising therapeutic strategy for diseases characterized by dysregulated cell death.

## Facts

- Ferroptosis, pyroptosis, and necroptosis are newly discovered forms of regulated cell death that regulate different pathophysiological processes.
- O-GlcNAcylation can modulate the activity of key proteins involved in ferroptosis, pyroptosis, and necroptosis, thereby influencing cell survival and death.
- Targeting O-GlcNAcylation, which serves as a key regulator in modulating novel forms of cell death holds great therapeutic potential and offers new strategies and directions.
- O-GlcNAcylation regulates the stability and activity of key proteins, acting as a double-edged sword in modulating ferroptosis, pyroptosis, and necroptosis.

## Open questions

- How can the specific molecular mechanisms by which O-GlcNAcylation regulates novel forms of regulated cell death, especially under different pathological conditions, be further elucidated?
- How to explain the dual role of O-GlcNAcylation in different diseases, which may act as both a protective mechanism and a contributor to disease progression in different pathological contexts?
- What are the potential therapeutic strategies for targeting O-GlcNAcylation in diseases characterized by dysregulated cell death?

## Introduction

Cell death is a critical process in maintaining homeostasis within biological systems, impacting cellular survival and functionality through various mechanisms [1]. Among these, novel regulated cell death (RCD) forms such as ferroptosis, pyroptosis, and necroptosis have garnered significant attention in recent years [2, 3]. These forms of cell death are distinct from classical apoptosis and are characterized by unique biochemical pathways and morphological changes. Ferroptosis is driven primarily by iron-dependent lipid peroxidation [4–6], while pyroptosis is associated with gasdermin-mediated membrane rupture [7–9], and necroptosis involves receptor-interacting protein (RIP) kinases [10–12]. Understanding the regulatory mechanisms governing these cell death pathways is essential, particularly in the context of disease states such as cancer, neurodegeneration, and inflammation.

O-GlcNAcylation is a reversible post-translational modification (PTM) catalyzed by O-GlcNAc transferase (OGT), which specifically attaches β-N-acetylglucosamine to the hydroxyl groups of serine (Ser) and threonine (Thr) residues, has emerged as a critical regulatory factor in diverse biological processes [13–15]. This modification is catalyzed by OGT and can be reversed by O-GlcNAcase (OGA) [16–18]. Recent studies reveal that O-GlcNAcylation plays a crucial role in modulating novel RCDs, particularly in ferroptosis, pyroptosis, and necroptosis [19, 20]. For instance, alterations in O-GlcNAcylation levels can influence the sensitivity of cells to ferroptosis, thereby affecting tumor cell viability and response to chemotherapy [21]. Furthermore, evidence indicates that O-GlcNAcylation may interact with other cellular signaling pathways, such as those involving reactive oxygen species (ROS) and lipid metabolism, to regulate the execution of ferroptosis [22, 23].

In the context of ferroptosis, O-GlcNAcylation has been shown to modulate the expression of key regulatory proteins involved in lipid peroxidation and antioxidant defense mechanisms [24]. For example, increased O-GlcNAcylation can enhance the activity of glutathione peroxidase 4 (GPX4) [25], a critical enzyme that protects against ferroptosis by reducing lipid hydroperoxides [26, 27]. Conversely, a decrease in O-GlcNAcylation may sensitize cells to ferroptosis, promoting cell death in various cancer models [28]. This highlights the potential of targeting O-GlcNAcylation as a therapeutic strategy in diseases characterized by dysregulated ferroptosis, such as cancer and neurodegenerative disorders.

Pyroptosis, characterized by its inflammatory nature, is also influenced by O-GlcNAcylation. Recent findings suggest that O-GlcNAcylation can modulate the activation of caspase-1, the enzyme responsible for cleaving gasdermin D (GSDMD) [29, 30], which forms pores in the cell membrane, leading to pyroptotic cell death [31]. The interplay between O-GlcNAcylation and pyroptosis underscores the complexity of cellular responses to stress and the potential for therapeutic interventions that manipulate these pathways.

Necroptosis, another form of RCD, is also subject to modulation by O-GlcNAcylation. The activation of necroptosis typically involves the formation of a necrosome complex containing receptor-interacting protein kinase 1 (RIPK1), receptor-interacting protein kinase 3 (RIPK3), and mixed-lineage kinase domain-like protein (MLKL), which orchestrates the cellular necrotic response [32, 33]. Emerging evidence suggests that O-GlcNAcylation can influence the signaling cascades leading to necroptosis, potentially by altering the stability and activity of key proteins within the necrosome [34]. The regulatory role of O-GlcNAcylation in necroptosis underscores its function in fine-tuning cellular survival-death balance, particularly under conditions of stress or injury.

Certain recent reviews have highlighted the role of O-GlcNAcylation in ferroptosis and pyroptosis [20, 35]. However, the specific contributions of O-GlcNAcylation in novel RCD within the context of diseases characterized by dysregulated cell death associated remain largely unexplored. This review aims to outline the functions of O-GlcNAcylation in ferroptosis, pyroptosis and necroptosis in diseases characterized by dysregulated cell death. Targeting O-GlcNAcylation presents a promising combined strategy for diseases characterized by dysregulated cell death therapy.

## O-GlcNAcylation

### Mechanism of GlcNAcylation

**Fig. 1:**
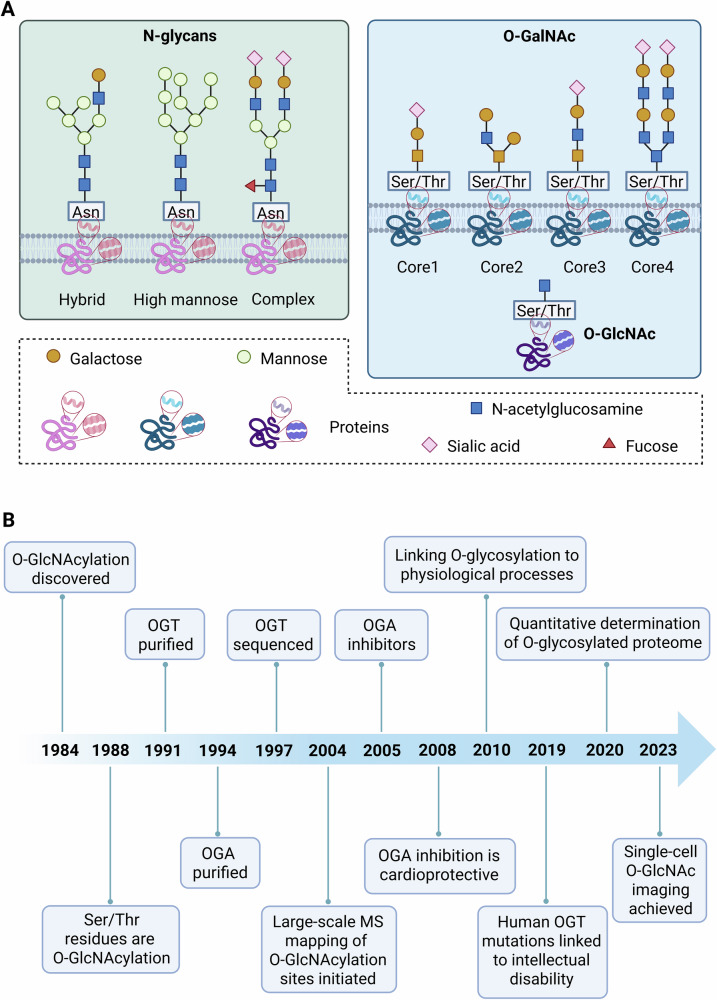
Mechanism of GlcNAcylation and timeline of O-GlcNAcylation. **A** Mechanism: This image illustrates the structural differences between N-glycosylation and O-glycosylation. N-glycosylation occurs on asparagine residues, forming complex glycan structures, whereas O-glycosylation occurs on Ser or Thr residues, forming structures like O-GlcNAc and O-GalNAc. **B** Timeline: The development of O-GlcNAcylation has seen significant progress from basic mechanisms to clinical applications. This timeline illustrates key advancements in O-GlcNAcylation research from the 1984 discovery of O-GlcNAc on Ser/Thr residues to the achievement of single-cell O-GlcNAc imaging in 2023.

Glycosylation is a crucial form of PTM that involves the addition of carbohydrate molecules to proteins [36, 37], thereby affecting their function and fate. Protein glycosylation is an enzymatically catalyzed PTM wherein carbohydrate structures, primarily oligosaccharides, form covalent linkages with specific amino acid side chains on target proteins. Based on the nature of the glycosidic bond and the specific amino acid residue involved, this modification is principally categorized into four types: O-glycosylation, N-glycosylation, C-glycosylation, and glycosylphosphatidylinositol (GPI)-anchored attachment [38, 39]. As depicted in Fig. 1A, glycosylation is primarily divided into N-glycans and O-glycans [39, 40]. N-glycans occur on asparagine (Asn) residues, forming complex glycan chain structures, including hybrid, high mannose, and complex types [41, 42]. These structures are typically composed of monosaccharides such as galactose, sialic acid, mannose, and fucose. O-glycans mainly occur on Ser or Thr residues [43, 44], forming structures such as O-linked-β-D-GlcNAc (O-GlcNAc) and O-acetylgalactosamine (O-GalNAc) [45, 46].

The mechanism of glycosylation involves the coordinated action of a series of enzymes, including glycosyltransferases and glycosidases, which are responsible for adding or removing glycan moieties on specific amino acid residues [47]. These modifications are essential not only for protein stability and folding but also involved in cellular signaling, cell recognition, and immune responses. In disease states, glycosylation patterns may change, such as the abnormal glycosylation commonly seen in cancer cells, which can affect tumor growth and metastasis [48]. Therefore, research on glycosylation not only helps us understand normal physiological processes but may also provide new targets for disease diagnosis and treatment. For example, by modulating the activity of glycosylation enzymes, one can influence protein function, thereby affecting cellular behavior, which has potential applications in drug development [45, 49].

### Development of O-GlcNAcylation

The study of GlcNAcylation has seen significant progress over the past few decades, particularly in the realm of O-GlcNAcylation (Fig. 1B). This modification involves the covalent attachment of GlcNAc to Ser or Thr residues of proteins, modulating their activity, stability, and intracellular localization. O-GlcNAcylation is catalyzed by OGT and reversed by OGA [50]. This dynamic PTM plays a crucial role in various cellular processes, including metabolism, signal transduction, and gene expression regulation [51].

Research into glycosylation began in the early 1980s when scientists started to explore the fundamental mechanisms of this modification. In 1988, the structure of GPI-anchored glycosylation was fully elucidated. In 1992, the initiating enzyme for GPI-anchored glycosylation, transamidase, was discovered. These early studies laid the foundation for subsequent research into glycosylation. Around 2000, the consensus sequence for N-glycosylation (Asn-X-Thr/Ser) and the structure of the Oligosaccharyltransferase complex subunits were identified. In 1984, protein Ser/Thr O-GlcNAcylation was first documented. Large-scale MS mapping of O-GlcNAc sites was launched in 2004, providing a technological foundation for functional studies. The first potent OGA inhibitors appeared in 2005, enabling targeted modulation of O-GlcNAc dynamics. Starting in 2008, OGA inhibition was demonstrated to be cardioprotective in ischemia–reperfusion models, bridging basic science and disease therapy. In the 2010s, the close links between O-glycosylation and various physiological processes, such as inflammation, immune escape, and viral infection, were revealed. O-GlcNAcylation plays a significant role in cell signaling, metabolic regulation, and gene expression [46]. For instance, O-GlcNAcylation can regulate the activity of cyclin-dependent kinases (CDKs), thereby influencing cell cycle progression. Additionally, O-GlcNAcylation is involved in regulating cellular stress responses, such as the activity of heat shock proteins (HSPs), helping cells maintain protein homeostasis under stress conditions. In 2019, human OGT mutations were linked to intellectual disability, establishing a direct clinical connection. Around 2020, quantitative determination of the O-glycosylated proteome was achieved. In 2023, single-cell O-GlcNAc imaging was achieved, offering new tools for mechanistic and translational research. These advancements have not only deepened our understanding of the biological functions of glycosylation but also provided new insights for disease diagnosis and treatment. In recent years, the role of O-GlcNAcylation in the regulation of cell death has garnered attention. Studies have shown that O-GlcNAcylation can modulate key proteins in various cell death pathways, thereby influencing cell survival and death [52].

### The functions of O-GlcNAcylation

Among O-glycosylations, O-GlcNAcylation is a dynamic PTM involving the covalent attachment of N-acetylglucosamine to Ser or Thr residues of proteins [53]. This modification is catalyzed by OGT, while OGA is responsible for removing the O-GlcNAc modification [54]. O-GlcNAcylation plays a significant role in various cellular processes, including metabolism, gene transcription, signal transduction and protein stability [55]. For instance, O-GlcNAcylation can regulate the activity of CDKs, thereby influencing cell cycle progression [56]. Additionally, O-GlcNAcylation is involved in regulating cellular stress responses, such as the activity of HSPs, helping cells maintain protein homeostasis under stress conditions [57].

In recent years, the role of O-GlcNAcylation in the regulation of cell death has come into focus. Studies have shown that O-GlcNAcylation can influence cell survival and death by modulating key proteins in various cell death pathways [58]. For example, O-GlcNAcylation modulates GPX4 activity to influence ferroptosis sensitivity [26], targets GSDMD activation to impact inflammatory responses during pyroptosis [31], and regulates RIPK1 activity to affect the necrotic process in necroptosis [59]. These findings collectively illuminate the intricate regulatory networks through which O-GlcNAcylation governs distinct cell death modalities. In the subsequent sections, we comprehensively summarize the functions and mechanisms of O-GlcNAcylation in regulating three novel RCDs, including ferroptosis, pyroptosis, and necroptosis (Table 1).

**Table 1:** The role of O-GlcNAcylation in novel regulated cell death.

Proteins	O-GlcNAcylation sites	Effect on its functions	Mechanisms	Effects	Sample types	Ref.
**Ferroptosis**						
SLC7A11	Ser26	O-GlcNAcylation promotes protein expression and transcriptional activity of SLC7A11	SLC7A11 O-GlcNAcylation leads to an increase in Fe^2+^ concentrations	SLC7A11 O-GlcNAcylation inhibits ferroptosis in hepatocellular carcinoma cells	Liver cancer	[68]
YAP	Thr241	O-GlcNAcylation enhances and stabilizes the expression of YAP	O-GlcNAcylation of YAP increases TFRC transcription and leads to elevated Fe^2+^concentration	Elevated YAP O-GlcNAcylation increases ferroptosis sensitivity	Hepatocellular carcinoma	[69]
ZEB1	Ser555	O-GlcNAcylation enhances the stability and nuclear translocation of ZEB1	O-GlcNAcylation of ZEB1 promotes the transcriptional activity of adipogenesis-related genes FASN and FADS2, leading to increased synthesis of PUFAs	O-GlcNAcylation of ZEB1 promotes ferroptosis in mesenchymal pancreatic cancer cells	Mesenchymal pancreatic cancer cells	[70]
YAP		Oh-Gelknassio enhances ap stability, nuclear translocation, and transcriptional activity	O-GlcNAcylation interferes with the ubiquitination of YAP K48 linking	YAP O-GlcNAcylation contributes to corneal epithelial cell ferroptosis under cigarette smoke exposure	Corneal epithelial cell	[71]
YAP	Thr241	O-GlcNAcylation antagonizes Ser127 phosphorylation to inhibit the degradation of YAP	Reduced O-GlcNAcylation inhibit the transcription of FTH1 by YAP, resulting in elevated LIP	Decreased O-GlcNAcylation of YAP increased ferroptosis sensitivity	Lung adenocarcinoma	[72]
c-Jun	Ser73	O-GlcNAcylation promotes protein expression, transcriptional activity and nuclear accumulation of c-Jun	O-GlcNAcylation of c-Jun stimulates GSH synthesis and reduces ROS accumulation	O-GlcNAcylation of c-Jun inhibits ferroptosis	Liver cancer	[73]
FTH	Ser179	De-O-GlcNAcylation of FTH promoted the degradation of FTH	De-O-GlcNAcylation of FTH increases the interaction with NCOA4 and promotes ferritinophagy, leading to elevated LIP	Inhibition of O-GlcNAcylation of FTH activates ferroptosis	U2OS cells, HUVEC and HT1080 cells	[19]
FTH	Ser179	HDN upregulates O-GlcNAcylated FTH protein in AML12 cells and inhibits FTH and NCOA4 interactions induced by DON exposure	De-O-GlcNAcylation of FTH increases NCOA4-mediated ferritin autophagy	De-O-GlcNAcylation of FTH activates ferritin autophagy and ferroptosis	Liver damage	[74]
TFRC	Ser687	Trafigura’s -oh-Gelknasilatio strong results in reduced ubiquitination of Trafigura and increased Teuca protein stability	De-O-GlcNAcylation of TFRC reduces the binding of ubiquitin E3 ligase membrane-associated RING-CH8 (MARCH8) and reduces polyubiquitination on Lys665	Tork’s Oh-Gelknassio controls ferroptosis in hepatocellular carcinoma cells	Hepatocellular carcinoma	[75]
Nrf2	Thr334	OGT promotes o-glcnacylation of Nrf2 at the binding site t334, which in turn promotes its nuclear translocation	O-GlcNAcylated Nrf2 binds to the G6PDH promoter region and increases G6PDH expression at both transcript and protein levels	O-GlcNAcylation attenuates I / R-induced iron death in lung epithelial cells through the nrf2/g6pdh axis	Lung epithelial cell death	[84]
HOXA9	Ser127	O-GlcNAcylation stabilizes HOXA9 and promotes its nuclear translocation in NPC cells	O-GlcNAc-modified HOXA9 promotes its mediated SIRT6 degradation by targeting ubr5 in NPC cells	When the O-GlcNAcylation modification of HOXA9 is reduced, the sensitivity of cells to iron death increases, thereby promoting cell death	Nasopharyngeal carcinoma	[28]
FOXK2	Ser424	O-GlcNAc modification enhances the nuclear localization ability of FOXK2	OGT modifies transcription factor FOXK2 through O-GlcNAc to enhance its transcription of SLC7A11	OGT activation drives the enhancement of O-GlcNAc signaling, and the activated ROS-OGT-FOXK2-SLC7A11 signaling axis inhibits ferroptosis	Hepatocellular carcinoma	[23]
NR3C1	Thr299	NR3C1 O-GlcNAcylation enhances protein stability and reduces proteasome dependent degradation by inhibiting ubiquitination	NR3C1 O-GlcNAcylation significantly enhances the transcriptional activity of GPX4 and regulates ferroptosis through the GFPT1/NR3C1/GPX4 axis	NR3C1 O-GlcNAcylation inhibits ferroptosis by promoting the binding of NR3C1 to the GPX4 promoter	Bladder cancer	[77]
METTL3	Ser118	O-GlcNAcylation of METTL3 can stabilize its expression	High expression of METTL3 promotes degradation of HMGB1 in an m6A-YTHDF2 dependent manner	O-GlcNAcylation with ubiquitination stabilizes METTL3 to inhibit ferroptosis	Pancreatic cancer	[78]
KGA	Thr563	O-GlcNAcylation of KGA can enhance enzyme activity and stability	GLS1 subtype KGA, utilizing glutamate derived from glutaminolysis, enhances GSH synthesis	O-GlcNAcylation of glutaminase isoform KGA inhibits ferroptosis	Hepatoblastoma	[79]
DJ-1	Thr19	OGT mediated O-GlcNAcylation of DJ-1 was crucial for maintaining its homodimeric structure.	O-GlcNAcylation-deficient mutation of DJ-1 enhanced the interaction between SAHH and the negative regulatory factor AHCYL1, thereby inhibited the activities of SAHH and transsulfuration pathway.	O-GlcNAcylation of DJ-1 inhibits ferroptosis	Renal cell carcinoma	[76]
KEAP1	Ser104	Reducing O-GlcNAc modification of KEAP1 promotes NRF2 nuclear translocation	FR054 enhanced temozolomide sensitivity by inhibiting protein O-GlcNAcylation via the upregulation of HMOX1 and downregulation of GPX4.	FR054 promotes ferroptosis in glioblastoma cells via KEAP1-NRF2-HMOX1 axis	Glioblastoma	[21]
**Pyroptosis**						
GSDMD	Ser338	O-GlcNAc modification of GSDMD results in reduced binding to caspases-4 in HEK293T cells	O-GlcNAc modification of GSDMD attenuates LPS-induced pyroptosis of endothelial cells by preventing its interaction with caspase-11 (a human homologous to caspases-4/5)	Gersdem’s Oh-Gerkenak modification attenuates Lipps-induced endothelial cell pyroptosis	Metastasizing septicemia	[31]
GSDME	Ser339	HG treatment enhanced OGT-mediated GSDME O-GlcNAcylation	GSDME interacts with OGT, and OGT knockdown inhibits O-GlcNAcylation of GSDME.	GSDME O-GlcNAcylatio enhances pyroptosis in LPS-induced macrophages.	Diabetic periodontitis	[94]
NLRP3	Thr542	LPS promotes O-GlcNAcylation of NLRP3 by enhancing OGT expression	LPS promotes pyroptosis of HGFs by enhancing OGT expression and promoting O-GlcNAcylation of NLRP3	O-GlcNAcylation of NLRP3 contributes to lipopolysaccharide-induced pyroptosis of human gingival fibroblasts	Human Gingival Fibroblasts	[95]
NLRP3		The O-GlcNAcylation of NLRP3 was elevated to enhance the stability of NLRP3 protein	BPA induces lipid metabolism dysfunction and pyroptosis by upregulating O-GlcNAc transferase levels	Increased levels of OH-Gelknac-transferase induce dysfunction of lipid metabolism and pyroptosis	NAFLD	[96]
NEK7	Ser260	Silencing OGT inhibits O-GlcNAcylation and enhances phosphorylation of NEK7 at S260	OGT-induced NEK7 O-GlcNAcylation promotes OA progression by promoting chondrocyte pyroptosis via the suppressing interaction between NEK7 and NLRP3	Silencing of OGT inhibited LPS-induced chondrocyte pyroptosis	Osteoarthritis	[97]
STAT3	Thr717	STAT3 O-GlcNAcylation enhances STAT3 phosphorylation	The absence of GSDME promotes OGT recruitment into the CUL4B-DDB1-WDR26 E3 ubiquitin ligase complex, reducing STAT3 O-GlcNAcylation	GSDME restricts cell pyroptosis by regulating O-GlcNAcylation and STAT3 S100A7A RAGE signaling pathways	Kidney diseases	[98]
NLRP3	Ser6	OGT mediated O-GlcN mediated NLRP3 promotes pyroptosis in PC12 cells	OGT inhibition downregulates the expression of NLRP3 and its O-GlcNAcylation	Downregulation of NLRP3 O-GlcNAcylation inhibits cell pyroptosis	Spinal cord injury	[100]
ATF2	Ser44	OGT mediated O-GlcNAcylation of ATF2 inhibits its phosphorylation and nuclear translocation	OGT mediated O-GlcNAcylation of ATF2 amplifies NLRP3 inflammasome activation	OTT mediated O-GlcNAcylation of ATF2 inhibits cell pyroptosis	Sepsis-associated encephalopathy	[99]
**Necroptosis**						
RIPK3	Ser/Thr	WMW inhibits the activation of RIPK1, RIPK3, and MLKL by increasing colonic O-GlcNAcylation levels	WMW enhances OGT activity and inhibits OGA activity, thereby increasing RIPK3 O-GlcNAcylation and inhibiting the binding of RIPK3 to MLKL	The traditional herbal formula Wu-Mei-Wan inhibits necroptosis by increasing RIPK3 O-GlcNAcylation	Colitis	[115]
RIPK3		SPC upregulates OGT-mediated O-GlcNAcylation, increases O-GlcNAcylated RIPK3, and inhibits the binding of RIPK3 to MLKL	The OGT inhibitor OSMI-1 abolishes SPC-mediated cardioprotective effects and inhibits OGT-mediated SPC-induced upregulation of O-GlcNAcylation and downregulation of RIPK3 and MLKL proteins	Sevoflurane post-treatment reduces necrotizing apoptosis induced by myocardial ischemia-reperfusion injury by upregulating OGT-mediated O-GlcNAcylated RIPK3	MIRI	[112]
RIPK3	Thr467	O-GlcNAcylation of RIPK3 at T467 inhibits its RHIM function	OGT-mediated O-GlcNAcylation of the serine-threonine kinase RIPK3 prevents RIPK3-RIPK1 heterologous and RIPK3-RIPK3 homogeneous interactions	O-GlcNAcylation of RIPK3 inhibits inflammation and necroptosis	HBP	[34]
RIPK1	Ser331	RIPK1 O-GlcNAcylation inhibits its interaction with RIPK3 in erythrocytes	O-GlcNAcylation of RIPK1 at human serine 331 (corresponding to serine 332 in mice) inhibits phosphorylation of RIPK1 at serine 166 and inhibits the formation of the RIPK1-RIPK3 complex in Ripk1 MEF	O-GlcNAcylation of RIPK1 rescues erythrocytes from necroptosis	Red blood cells	[59]

## The role of O-GlcNAcylation in novel regulated cell death

### O-GlcNAcylation and Ferroptosis

#### Ferroptosis

Ferroptosis represents a unique type of RCD driven by iron-dependent accumulation of lipid peroxides, ultimately reaching toxic levels that compromise cellular integrity [60]. Unlike apoptosis, which is a well-known programmed cell death pathway [61], ferroptosis is defined by its unique biochemical and morphological features that include mitochondrial shrinkage and increased membrane density [62, 63]. The process is primarily driven by oxidative stress, leading to the accumulation of lipid hydroperoxides, which ultimately disrupt cellular integrity and function [64]. Key players in the regulation of ferroptosis include the System Xc- glutathione (GSH) pathway [65, 66], which is crucial for maintaining cellular redox homeostasis, and GPX4, an enzyme that detoxifies lipid peroxides [67]. Disruption of these pathways can trigger ferroptosis, making it a significant area of research in various pathologies, including cancer and neurodegenerative diseases [22, 26]. The understanding of ferroptosis has evolved over the years, revealing its implications in tumor suppression and the therapeutic potential of inducing ferroptosis in cancer cells, particularly in those resistant to conventional treatments.

#### O-GlcNAcylation in ferroptosis

**Fig. 2:**
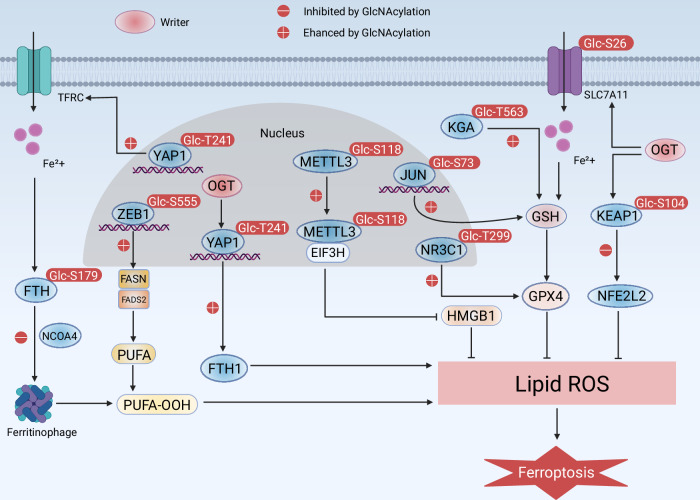
O-GlcNAcylation in ferroptosis. O-GlcNAcylation mediated by OGT plays a crucial role in regulating ferroptosis through modifying ferritin, YAP1, ZEB1, c-Jun, FTH, SLC7A1, METTL3, NR3C1, KEAP1, and KGA. Ferroptosis is a non-apoptotic form of cell death characterized by overwhelming membrane lipid peroxidation and iron accumulation. TFRC primarily promotes ferroptosis by transferring Fe^2+^ into cells, and NCOA4-mediated ferritin autophagy may play a role in regulating cellular iron levels by targeting ferritin for degradation. Subsequently, Fe^2+^ generates ROS through the Fenton reaction. The promotion or inhibition of protein function by O-GlcNAcylation is indicated by a positive (+) or negative (-) sign.

O-GlcNAcylation represents a rapidly evolving regulatory mechanism that critically modulates ferroptosis through multifaceted interactions with the system Xc − , specific transcription factors, and autophagic flux (Fig. 2). OGT O-GlcNAcylates SLC7A11 at Ser26, a key modification that regulates the cystine transport activity of SLC7A11 [68]. O-GlcNAc modification at Thr241 on YAP1 and Ser555 on ZEB1 enhanced their transcriptional activity and stability [69, 70]. Consequently, elevated glucose concentrations may enhance cellular susceptibility to ferroptosis by facilitating the O-GlcNAcylation of YAP1 and ZEB1 in hepatocellular carcinoma (HCC) and pancreatic cancer cells [69, 70]. Another study revealed that the O-GlcNAcylation of YAP1 interfered with the ubiquitination of YAP K48 linkage [71]. In addition, de-O-GlcNAcylation of YAP1 at Thr241 increased ferroptosis susceptibility [72]. Therefore, it is possible that hyperglycemia levels may increase susceptibility to ferroptosis by promoting de-O-GlcNAcylation of YAP1 in lung adenocarcinoma cells. However, O-GlcNAcylated c-Jun hinders ferroptosis by promoting GSH synthesis in HCC [73]. In addition, de-O-GlcNAcylation of ferritin FTH at Ser179 enhanced its interaction with the ferritin autophagy receptor NCOA4 [19, 74]. Specifically, pharmacological or genetic repression of O-GlcNAcylation promotes ferritin autophagy and mitophagy, inducing ferroptosis by increasing the accumulation of unstable iron in U2OS cells, HUVEC, and HT1080 cells [19]. Moreover, TFRC controls ferroptosis in HCC cells by O-GlcNAcylation at the site Ser687 [75]. In addition to TFRC, emerging evidence shows that, ROS-activated OGT suppresses ferroptosis through O-GlcNAcylation of FOXK2 and DJ-1 in certain cancers [23, 76]. O-GlcNAcylation promotes tumor progression and therapy resistances across various cancer types through distinct mechanisms, including enhancing GPX4 expression [77], inducing drug resistance [78], and boosting GSH synthesis [79]. Moreover, O-GlcNAcylation of KEAP1 at Ser104 facilitates the ubiquitin-dependent proteasomal degradation of NFE2L2, a critical transcription factor that suppresses ferroptosis, in response to fluctuations in glucose availability [80, 81]. These results establish a functional connection between dynamic O-GlcNAcylation and the regulation of ferroptosis, O-GlcNAcylation regulates ferroptosis through site-specific modifications of key targets, although the exact crosstalk mechanism between iron autophagy and mitophagy needs to be further elucidated.

The relationship between O-GlcNAcylation and ferroptosis is mediated through several signaling pathways, with the Nuclear factor erythroid 2-related factor 2 (Nrf2) -ARE pathway being particularly significant. Nrf2 is a transcription factor that plays a crucial role in cellular defense against oxidative stress [82, 83]. Research has demonstrated that O-GlcNAcylation can enhance the activity of Nrf2, leading to the upregulation of various antioxidant genes, including those involved in GSH synthesis [84]. This pathway not only helps in maintaining cellular redox balance but also contributes to ferroptosis resistance by promoting the expression of protective enzymes like GPX4. Notably, the reciprocal regulation between O-GlcNAcylation and SLC7A11-xCT system further fine-tunes cellular susceptibility to ferroptosis, as evidenced by lipid peroxidation assays [84]. These findings underscore the complexity of the signaling networks that connect O-GlcNAcylation and ferroptosis, offering potential avenues for therapeutic intervention in diseases characterized by dysregulated cell death mechanisms.

### O-GlcNAcylation and pyroptosis

#### Pyroptosis

Pyroptosis is a type of regulated cell death defined by gasdermin-mediated pore formation in the plasma membrane [85], resulting in osmotic lysis and the extensive secretion of pro-inflammatory mediators [86]. It is primarily mediated by a family of proteins known as gasdermins [87, 88], which play a crucial role in the immune response to infections and inflammation [89]. Upon activation, gasdermins undergo proteolytic cleavage, which liberates their N-terminal domains that can oligomerize and form pores in the plasma membrane [90]. This pore formation results in the uncontrolled release of cellular contents, including inflammatory mediators, which can exacerbate tissue damage and further propagate the inflammatory response [91]. Pyroptosis is distinct from apoptosis and necroptosis, as it is specifically associated with inflammation and is often triggered by pathogenic infections or cellular stressors [92, 93]. The understanding of pyroptosis has expanded significantly, revealing its implications in various diseases, including sepsis, cancer, and autoimmune disorders.

#### O-GlcNAcylation in pyroptosis

**Fig. 3:**
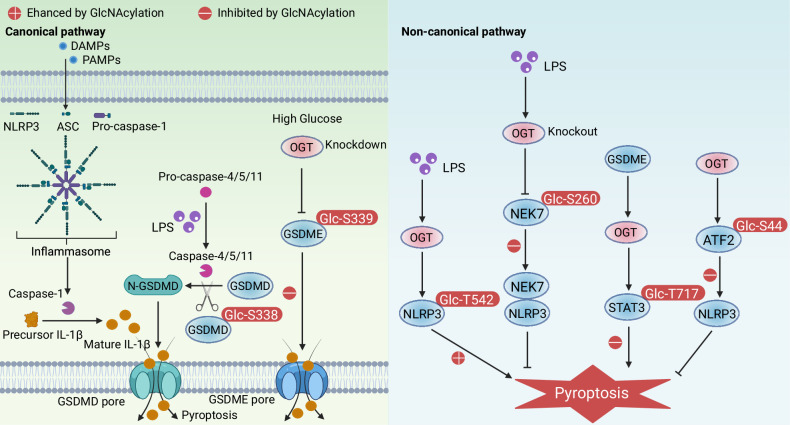
O-GlcNAcylation in pyroptosis. O-GlcNAcylation mediated by OGT plays a crucial role in regulating pyroptosis through modifying GSDMD, GSDME, NLRP3, NEK7. The molecular mechanisms of pyrolysis mainly include canonical and noncanonical signaling. PAMPs and DAMPs interact with cytosolic sensor proteins through a typical pathway. Then inflammasome assembly activates caspase-1, activates IL-1β, forms N-GSDMD, releases IL-1β through GSDMD pores, and promotes oligomeric NINJ1. LPS activates caspase-4/5/11, leading to GSDMD cleavage and GSDMD pore formation, resulting in potassium efflux, and LPS can also trigger NLRP3 inflammasome and lead to pyroptosis. GSDME interacts with OGT, and OGT knockdown inhibits O-GlcNAcylation of GSDME in the environment of high glucose. OGT-induced NEK7 O-GlcNAcylation promote chondrocyte pyroptosis via the suppressing interaction between NEK7 and NLRP3.

O-GlcNAcylation acts as a multifaceted regulator of pyroptosis, targeting key signaling molecules and inflammasome components across both canonical and non-canonical pathways, and critically influencing inflammasome activation and gasdermin protein functions (Fig. 3). In the canonical pathway, O-GlcNAc modification at Ser338 on GSDMD disrupts its molecular interaction with caspase-11 (the functional human homolog of caspases-4/5), thereby mitigating LPS-induced endothelial cell damage [31]. In addition, OGT knockdown inhibits the O-GlcNAcylation of GSDME at Ser339 in a high glucose environment, and knockout of OGT inhibits pyroptosis in high glucose-treated macrophages, while GSDME overexpression partially reverses this inhibition [94]. Therefore, hyperglycemia levels have the potential to increase susceptibility to pyroptosis by promoting O-GlcNAcylation of GSDME in diabetic periodontitis cells [94]. In the non-canonical pathway, the expression of NLRP3 was enhanced by O-GlcNAcylation at Thr542 [95, 96]. Thus, LPS-induced O-GlcNAcylation of NLRP3 in human gingival fibroblasts increases susceptibility to pyroptosis [95]. However, OGT-induced O-GlcNAcylation of NEK7 at Ser260 promotes chondrocyte pyroptosis by inhibiting the interaction between NEK7 and NLRP3 [97]. O-GlcNAcylation exerts cell type- and context-dependent effects on pyroptosis, attenuating pyroptosis in nephrotoxicity and neuroinflammation via STAT3 and ATF2 [98, 99], respectively, while promoting pyroptosis in spinal cord injury through NLRP3 [100]. These findings reveal a novel link between dynamic O-GlcNAcylation and pyroptosis.

Several factors can influence the process of pyroptosis, with metabolic state and the activity of O-GlcNAc enzymes being paramount. The cellular metabolic environment can dictate the levels of O-GlcNAcylation, which in turn affects the activation of gasdermins and the subsequent pyroptotic response. For instance, high glucose availability has been associated with increased O-GlcNAcylation, which can enhance the activity of glycolytic enzymes and promote a pro-inflammatory state conducive to pyroptosis [101, 102]. Additionally, the activity of OGA is critically involved in this process, as its inhibition results in elevated O-GlcNAc levels that may promote pyroptotic signaling [103]. The balance between OGT and OGA activities is crucial for maintaining cellular homeostasis and regulating cell death pathways [104]. Overall, understanding these factors is vital for advancing therapeutic strategies and improving pyroptosis disease management, particularly those characterized by excessive inflammation.

### O-GlcNAcylation and necroptosis

#### Necroptosis

Necroptosis is a form of programmed cell death that is closely associated with inflammatory responses [105]. Unlike apoptosis, which is characterized by cell shrinkage and membrane blebbing [106, 107], necroptosis results in cell swelling and rupture [108], leading to the release of cellular contents into the extracellular space [109]. This process is mediated by a complex of proteins including RIPK1, RIPK3, and MLKL [110, 111]. The activation of RIPK1 is crucial for initiating necroptosis, as it forms a signaling complex with RIPK3 that ultimately leads to MLKL oligomerization and membrane permeabilization [112]. The inflammatory response triggered by necroptosis is significant, as the release of damage-associated molecular patterns from necroptotic cells can activate immune cells and promote further inflammation [113]. This has been observed in various pathological conditions, including neurodegenerative diseases, infections, and cancer, where necroptosis contributes to tissue damage and inflammation [32, 114]. Moreover, necroptosis has been implicated in the pathogenesis of diseases such as inflammatory bowel disease and acute respiratory distress syndrome, highlighting its dual role in both promoting inflammation and serving as a defense mechanism against pathogens.

#### O-GlcNAcylation in necroptosis

**Fig. 4:**
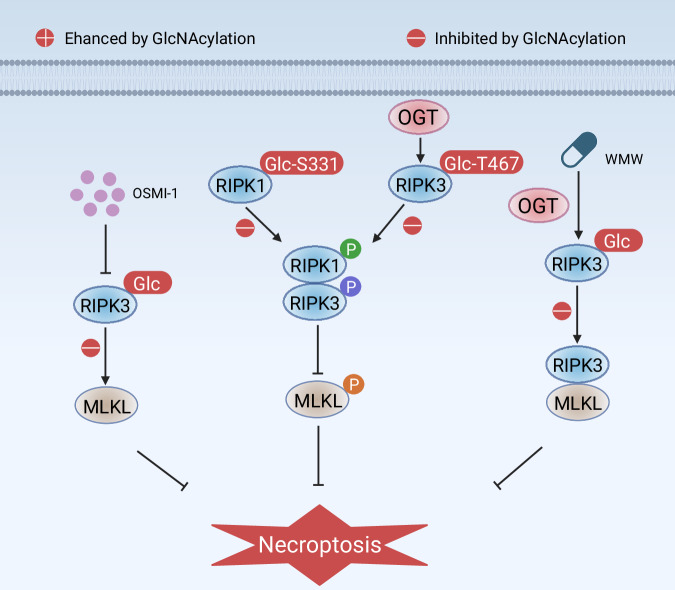
O-GlcNAcylation in necroptosis. O-GlcNAcylation mediated by OGT plays a crucial role in regulating necroptosis through modifying RIPK3 and RIPK1. O-GlcNAcylation of RIPK1 at human Ser 331 (corresponding to Ser 332 in mice) inhibits phosphorylation of RIPK1 at Ser 166 and inhibits the formation of the RIPK1-RIPK3 complex in RIPK1 MEF. In addition, the O-GlcNAcylation of RIPK3 at the T467 site inhibits its RHIM function, preventing the heterologous activity of RIPK3-RIPK1. The OGT inhibitor OSMI-1 inhibits OGT-mediated SPC-induced upregulation of O-GlcNAcylation and downregulation of RIPK3 and MLKL proteins. WMW (the pills) enhances OGT activity and inhibits OGA activity, thereby increasing RIPK3 O-GlcNAcylation and inhibiting the binding of RIPK3 to MLKL.

O-GlcNAcylation exerts a context-dependent regulatory role in necroptosis by targeting key regulatory proteins such as RIPK1 and RIPK3, thereby influencing necroptotic execution across various pathological conditions (Fig. 4). O-GlcNAcylation of RIPK3 inhibits the binding of RIPK3 to MLKL [112, 115]. Thus, O-GlcNAcylation of RIPK3 reduces necrotizing apoptosis induced by colitis and myocardial ischemia-reperfusion injury [112, 115]. In addition, O-GlcNAcylation of RIPK3 at site Thr467 enhances the stability of NLRP3 protein and induces lipid metabolism dysfunction and pyroptosis, leading to the production of nonalcoholic fatty liver disease [34]. However, O-GlcNAcylation of RIPK1 at Ser331 inhibits its interaction with RIPK3 in erythrocytes [59]. Thus, O-GlcNAcylation of RIPK1 saves erythrocytes from necroptosis [59]. These findings reveal a novel link between dynamic O-GlcNAcylation and necroptosis.

The clinical relevance of necroptosis and its regulation by O-GlcNAcylation is increasingly recognized in various diseases. Necroptosis has been implicated in the pathogenesis of conditions such as cancer, where it can influence tumor progression and response to therapy [116]. For example, necroptosis can promote inflammation within the tumor microenvironment, potentially aiding in tumor immune evasion and metastasis [117]. Furthermore, the modulation of O-GlcNAcylation has been proposed as a novel therapeutic target in diseases characterized by excessive necroptosis, such as inflammatory bowel disease and neurodegenerative disorders [59, 115, 118]. By understanding the regulatory mechanisms of O-GlcNAcylation in necroptosis, researchers aim to develop strategies that could enhance or inhibit this form of cell death, thereby improving therapeutic outcomes in conditions where necroptosis is a key pathogenic driver. Overall, the intersection of O-GlcNAcylation and necroptosis presents a promising area for future research, particularly in the context of chronic inflammatory diseases and cancer.

## Research prospects of O-GlcNAcylation in novel regulated cell death

### Clinical potential

O-GlcNAcylation levels show significant potential as an emerging biomarker for clinical diagnostics, with its alteration being associated with various disease states. Currently, several clinical trials are investigating its therapeutic utility. Several ongoing studies use O-GlcNAcylation levels as a key biomarker or intervention target. For example, NCT06754709 and NCT06754696 are recruiting pediatric patients to examine the role of O-GlcNAcylation in disease prevention and treatment (Table 2). For the latest information, please refer to the clinicaltrials.gov database. The development of treatment strategies centered on O-GlcNAcylation shows considerable promise for managing conditions such as sepsis. It is noteworthy that the current clinical research on O-GlcNAcylation is not associated with to RCD and remains largely at the preclinical stage, with no completed clinical trials reported to date.

**Table 2:** Clinical trials exploring the application of O-GlcNAcylation in diseases.

ClinicalTrials.gov identifier	Study title	Status	Disease type	Intervention/treatment	Study Type
NCT06754696	Evaluation of Blood Protein O-GlcNAcylation Levels in Children	Recruiting	Sepsis	None	Observational
NCT06754709	Evaluation of Blood and Cardiac Protein O-GlcNAcylation Levels in Cardiac Surgery in Children	Recruiting	Heart Diseases	None	Observational
NCT04440566	O-GlcNAcylation Role in the Pathophysiology of Systemic Lupus Erythematosus	Completed	Systemic Lupus Erythematosus	Biological: blood sample	Interventional
NCT05725005	PET Study of Repeated ASN51 in Healthy Volunteers	Completed	Alzheimer disease	Drug: ASN51	Interventional

*RIP* receptor-interacting protein; *PTM* post-translational modification; *Ser* serine; *Thr* threonine; *OGT* O-GlcNAc transferase; *OGA* O-GlcNAcase; *ROS* reactive oxygen species; *GPX4* glutathione peroxidase 4; *GSDMD* gasdermin D; *RIPK1* receptor-interacting protein kinase 1; *RIPK3* receptor-interacting protein kinase 3; *CDKs* cyclin-dependent kinases; *HSPs* heat shock proteins; *Asn* asparagine; *GlcNAc* N-acetylglucosamine; *GSH* glutathione; *Nrf2* nuclear factor erythroid 2-related factor 2; *GSH* glutathione; PUFA polyunsaturated fatty acid; *FASN* fatty acid synthase; *FADS2* fatty acid desaturase 2; *c-Jun* activator protein-1; *YAP* Yes-associated protein; *ZEB1* zinc finger E-box-binding homeobox 1; *FTH* ferritin heavy chain; *SLC7A11* solute carrier family 7 member 11; *Lipid ROS* lipid-reactive oxygen species; *Glc* Glycosylation; *O-GalNAc* O-acetylgalactosamine; *O-GlcNAc* O-linked-β-D-GlcNAc; *LPS* lipopolysaccharide; *IL-1β* interleukin-1; *GSDMD* GasderminD; *GSDME* GasderminE; *NEK7* Never in mitosis gene A-related kinase 7; *NLRP3* NOD-like receptor protein 3; *Caspase* Cysteine-aspartic acid protease; *MLKL* mixed-lineage kinase domain-like protein; *PAMPs* pathogen-associated molecular patterns; *DAMPs* damage-associated molecular patterns; *HCC* hepatocellular carcinoma; *NPC* nasopharyngeal carcinoma.

Although clinical studies on O-GlcNAcylation and ferroptosis, pyroptosis and necroptosis are lacking, preclinical evidence reveals therapeutic potential value in various diseases. Preclinical research has shown that O-GlcNAcylation plays a significant role in the regulation of ferroptosis in HCC and nasopharyngeal carcinoma (NPC). In HCC, pharmacological inhibition or knockout of USP8 reduces the stability of OGT, thereby suppressing HCC progression and inducing ferroptosis, indicating that targeting USP8 is a potential therapeutic strategy for HCC [68]. In NPC, O-GlcNAcylated HOXA9 enhances the expression of UBR5 and the ubiquitination and degradation of SIRT6, knockdown of HOXA9 or UBR5 promotes ferroptosis and inhibits NPC growth in mice, which provides a potential therapeutic target for NPC treatment [28]. Furthermore, chronic temozolomide exposure activates the HBP-PGM3 axis to elevate O-GlcNAcylation, which inhibits ferroptosis in glioblastoma; this chemoresistance can be reversed by targeting PGM3, thereby reactivating ferroptosis and suppressing tumor growth [21]. In addition, O-GlcNAcylation also has important clinical value in the regulation of pyroptosis in osteoarthritis. A recent study has shown that OGT-induced NEK7 O-GlcNAcylation levels were increased in both osteoarthritis and its experimental models, and knockout of OGT could alleviate osteoarthritis progression in model mice [97]. However, the contribution of O-GlcNAcylation to necroptosis-associated disease progression warrants further investigation using genetic manipulation models. Although no clinical studies on O-GlcNAcylation and cell death modalities have been conducted yet, the in-depth exploration of preclinical research offers valuable directions and insights for the design and implementation of future clinical trials. With continuous technological advancements and research progress, O-GlcNAcylation is emerging as a promising therapeutic target for neurological disorders, cancers, and metabolic diseases, offering potential for precise intervention in disease-specific signaling pathways.

### Future perspectives

Future research on O-GlcNAcylation should focus on several critical areas to fully harness its therapeutic potential in cancer and other diseases. First, elucidating the specific mechanisms by which O-GlcNAcylation influences cell death processes, such as ferroptosis, pyroptosis, and necroptosis, is essential. This includes understanding how O-GlcNAcylation modulates key proteins and pathways that mediate these cell death programs. For example, recent studies have shown that O-GlcNAcylation can affect the stability and activity of proteins like SLC7A11 in ferroptosis and p53 in pyroptosis, highlighting the need for more detailed mechanistic studies [68].

Second, investigating the crosstalk between O-GlcNAcylation and other PTMs, such as phosphorylation [119], ubiquitination, and acetylation, is crucial. These modifications often work in concert to regulate cellular responses to stress and damage [120]. Understanding their interplay could reveal how O-GlcNAcylation integrates into broader cellular signaling networks and how it can be targeted therapeutically.

Third, emerging technologies such as CRISPR-Cas9 gene editing [121], mass spectrometry [122], and single-cell sequencing enables the dissection of O-GlcNAcylation effects on specific substrates across diverse cellular contexts [123]. Using the CRISPR-Cas9 knock in strategy to mutate O-GlcNAcylation sites into Ala or Glu phosphate/glycosylation mutants, and verify their effects on the assembly of necrosomes [124]. Additionally, using click chemistry based proximity ligation in situ hybridization to analyze the co-localization of lipid peroxidation/pyroptosis regions on tissue slices [125]. These approaches will facilitate the discovery of novel targets and associated pathways for therapeutic intervention in diseases driven by aberrant O-GlcNAcylation.

Finally, translational studies should focus on developing and testing inhibitors of O-GlcNAcylation enzymes, such as OGT and OGA, in preclinical models of cancer and inflammatory diseases. For instance, OGT-inhibitor crystal structures facilitate the design of highly selective OGT inhibitors [126]. AI-driven platforms like ED2Mol enable de novo molecular generation by integrating electron density and binding pocket data, aiding in discovering novel allosteric or orthosteric inhibitors [125]. Additionally, natural products (e.g., tartary buckwheat antioxidant peptides) offer unique scaffolds for developing OGT and OGA inhibitors [127]. These studies should aim to validate the therapeutic efficacy and safety of such compounds, paving the way for clinical trials.

## Conclusions

The intricate role of O-GlcNAcylation in various forms of cell death, including ferroptosis, pyroptosis, and necroptosis, underscores its significance in cellular physiology and pathology. As this review highlights, O-GlcNAcylation serves as a critical PTM that modulates key signaling pathways involved in the regulation of these death processes. Understanding the molecular mechanisms by which O-GlcNAcylation influences cell fate decisions is essential for developing innovative therapeutic strategies aimed at mitigating the effects of related diseases.

The development of molecular mechanisms in O-GlcNAcylation is marked by a growing recognition of the complexity of cell death regulation and the need for a nuanced approach to research. The interplay between O-GlcNAcylation and various signaling cascades presents both challenges and opportunities for researchers. It is crucial to balance perspectives from different research findings, as some studies may highlight the protective roles of O-GlcNAcylation while others may suggest its involvement in promoting cell death in specific contexts [128, 129]. Furthermore, O-GlcNAcylation also bidirectionally regulates other RCDs. For example, it regulates apoptosis via AKT pathway and STAT3/FOXO1 axis in a stress-context-dependent manner [130, 131], while divergently controlling autophagy-mediated cell fate, promoting chondrocyte death by impairing GATA4 clearance while enhancing β-cell survival via mTORC1 activation [132, 133]. This duality necessitates a careful analysis of experimental conditions, biological models, and the broader physiological context in which these processes occur.

Future research should aim to delineate the precise mechanisms by which O-GlcNAcylation affects the modulation of cell death pathways. This includes investigating the dynamic regulation of O-GlcNAc levels in response to different pathological stimuli, as well as identifying the downstream effectors that mediate its effects on cell survival or demise. Moreover, integrating findings from diverse fields such as cancer biology, neurobiology, and immunology will be key to constructing a comprehensive understanding of how O-GlcNAcylation can be targeted for therapeutic intervention.

Nevertheless, deciphering the complex mechanisms of O-GlcNAcylation within the framework of novel RCD pathways presents substantial research challenges. First, a single protein (e.g., RIPK1) may undergo various PTMs which can coordinately regulate novel RCDs. O-GlcNAcylation of RIPK1 suppresses necroptosis by inhibiting phosphorylation and indirectly modulates pyroptosis by altering RIPK1 conformation or interaction interfaces [134]; whereas direct evidence is currently lacking, this PTM may also indirectly regulate ferroptosis, given the established roles of RIPK1 in ROS generation and mitochondrial dysfunction [135]. Achieving precise control over these PTMs and their downstream cell death pathways remains difficult, thereby driving the need for innovative and precisely targeted therapeutic approaches. Second, current research is intensively investigating the roles of certain well-established modifications (such as N-glycosylation) in disease pathogenesis. Exploring the links between N-glycosylation and novel RCDs is one of the future research directions. However, existing analytical techniques may be insufficient to comprehensively characterize all aspects of protein glycosylation, particularly at the in vivo level. Accurate detection and quantification of site-specific N-glycan occupancy remain technically challenging with current methodologies, potentially limiting advances in glycobiology. Third, the clinical translation of O-GlcNAcylation, particularly in cell death, trails significantly behind other PTMs. Compared with phosphorylation, acetylation, and ubiquitination/SUMO that have already made breakthroughs in clinical practice (such as imatinib [136], HDAC inhibitors [137], lenalidomide [138]), the O-GlcNAcylation exhibits markedly delayed clinical progression. Future research should focus on the development of safe and effective intervention tools, thoroughly elucidate their pathological mechanisms and clinical value, in order to fully unleash the enormous potential of O-GlcNAc as a therapeutic target and biomarker, and achieve a leap from bench to bedside.

In conclusion, the exploration of O-GlcNAcylation represents a promising avenue for enhancing our understanding of cell death mechanisms and their implications in disease [46, 139]. By fostering interdisciplinary collaboration and encouraging the integration of various research perspectives, paving the way for the development of novel therapeutic strategies, such as immunotherapy [140, 141], that harness the regulatory potential of O-GlcNAcylation to improve clinical outcomes in diseases characterized by dysregulated cell death.
